# Making chemistry accessible for learners with vision impairment

**DOI:** 10.1038/s42004-023-01033-x

**Published:** 2023-10-27

**Authors:** Zoe Schnepp, Robyn Watson

**Affiliations:** 1 School of Chemistry, University of Birmingham, Birmingham, B152TT UK; 2 Bolton Sensory Support Service, Thomasson Memorial School, Devonshire Road, Bolton, BL1 4PJ UK

**Keywords:** Chemical education, Communicating chemistry

## Abstract

Pupils with vision impairment face significant challenges in learning science. Here, the authors discuss the impact of an inaccessible curriculum and new ideas that can improve accessibility.

Chemistry is a very visual subject. As professional chemists we take for granted the ability to understand detailed figures and graphs. In the laboratory, we rely on our eyesight for even the simplest procedures such as weighing out reagents and monitoring reactions. We also use specialist instruments that require precise alignment and operation. Even when we are familiar with the complex concepts of our field, it’s hard to imagine how we would manage to engage with chemistry without being able to see.

In England, in the 2020/21 academic year, there were 13,328 pupils recorded as having vision impairment (VI) as their primary special educational need (SEN)^1^. Of these, 9822 have VI as their only SEN. Pupils with VI are typically educated in mainstream schools and are often paired with a sighted partner for science experiments. Having no active role in the experiment can limit their ability to access learning and reduce interest in the subject. Students may have a dedicated classroom assistant, but a lack of confidence in STEM subjects can sometimes limit what the assistant offers. The teaching of scientific concepts is often achieved through diagrams, which can be inaccessible for pupils with VI, without adaptation and specialist description. Pupils are able to grasp scientific concepts as readily as their sighted peers, but their learning is impaired by a lack of accessibility and a need for more time to explore new concepts.

If lessons are inaccessible, students fall behind in their educational attainment. Across all school years in England, children with VI have been shown to be significantly behind their sighted peers in attainment in English, maths and science^1^. This has an impact on aspirations and wellbeing and, in turn, on success in further education or employment. For example, in the UK in 2015, 42.8% of people with VI aged 16–25 were not in employment, education or training^2^ compared to 11.7% of all 16–25 year olds^3^. Similar issues are faced by young people with VI in other countries^4^. It is important to note that missing out on practical science at school also impacts learning more widely. Science experiments teach skills such as critical thinking and problem solving, which are important more broadly in life^5^.

Most chemists working in higher education will not have encountered students with a VI. This is for the simple reason that many students with VI are put off STEM while still at school, as they feel that they are subjects they cannot engage with. Designing a curriculum that is accessible requires a great deal of creativity and ingenuity. These are skills that chemistry researchers have in abundance, so we are the ideal community to address this challenge.

## General strategies to improve accessibility

With increasing international attention on accessibility, some strategies may already be familiar. Using Alt text on images and captions or descriptions on videos is a simple way to make your website or learning resource more accessible to people with VI. Colour and contrast are also important. Many people with VI have some useable vision and a careful choice of colours can make a big difference to accessibility. In graphs and figures, consider using text, symbols and patterns alongside colour, whilst also making gridlines and scales simple and bold^6^.

In school laboratories, accessibility can be improved in surprisingly simple ways. Tactile stickers of different sizes and shapes can be used for labelling. Braille label makers are also available, although these may have limited reach, as many pupils access learning using large print or screen readers. Plastic syringes can be modified with a notch in the barrel to measure specific volumes of liquid^7^. This is something that can be done quickly and cheaply by a school technician, showing that modifications are often thoughtful tweaks rather than expensive purchases. Audio technology is also available, for example talking thermometers, weighing scales or colour detectors. However, it’s also important to consider that adaptive technology can give rise to feelings of ‘otherness’ for a pupil with VI in a mainstream school and so may not be appropriate; it can also cause issues in a noisy classroom, where things may not be heard clearly.

## Tactile models

Tactile models can be very effective in illustrating scientific concepts. One example is Tactile Collider, which was designed to make particle accelerator physics accessible to people with VI^8^. Pupils who took part in the project said that it inspired them to learn new things. More importantly, pupils who participated said the experience made them more confident to ask for modifications in school if they felt something was inaccessible. They also said it showed them that further study in the sciences was something they could aspire to.

**Fig. 1:**
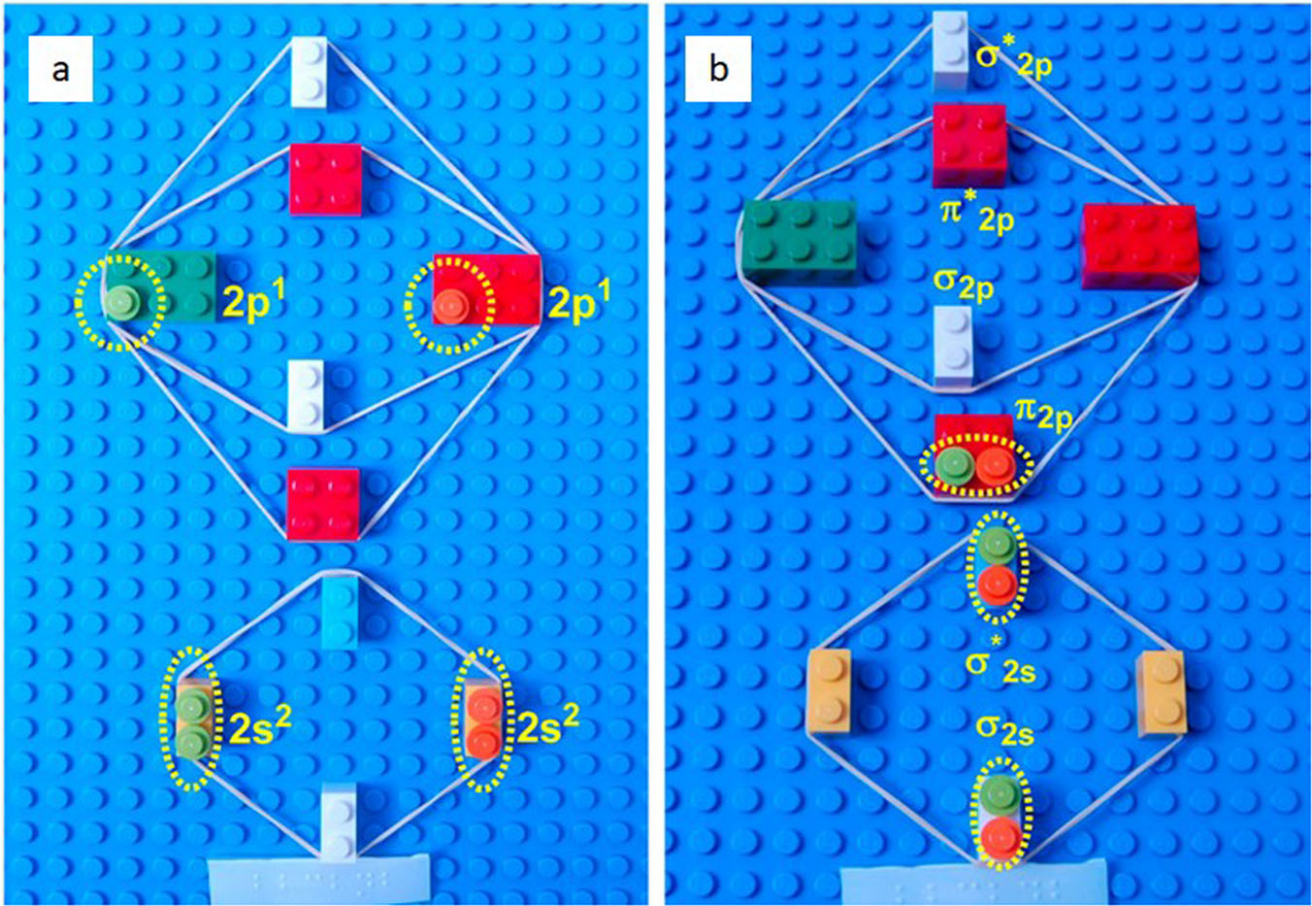
Model of the molecular orbital diagram of a B_2_ species made with interlocking toy bricks. Small round bricks are used to represent the electrons in (**a**) the atomic orbitals and (**b**) the molecular orbitals. A model like this would be used alongside a verbal description. Image reproduced from ref. ^10^.

Another simple example of a tactile demonstration is a building block model of a lithium battery^9^. The wooden pieces of the tower are decorated to represent the oxide and graphite electrodes and the lithium ions. The lithium ion pieces can then be transferred from one tower to gaps in a second tower to represent the charging and discharging processes. Plastic construction bricks have also been used to illustrate concepts such as periodic trends and even molecular orbital theory (Fig. 1)^10^. An important point to note about tactile models is that they should be carefully designed and tested with a VI audience in mind. It’s easy to make a tactile version of any scientific diagram but while something may seem obvious to a sighted designer it may not translate well into a tactile form. Complex structures and extensive details can become blurred, and the details lost when models or diagrams are too intricate. This can result in a model which is confusing for a pupil with VI. It is also important to note that models which are simple will still require some level of description in order to allow students to visualise the concepts being delivered.

## Experiments using other senses

Much of chemistry education involves experiments and it can be very challenging to make these accessible for pupils with VI. However, some ingenious solutions have been found that rely on senses other than sight to monitor chemical processes and reactions. A fascinating example is the use of onions to detect the endpoint of a titration^11^. Sodium hydroxide inhibits the formation and release of pungent sulfur compounds from onions and neutralisation of the solution with hydrochloric acid releases a strong onion odour. This experiment can be easily adapted for pupils with VI^12^ and provides an interesting (albeit smelly) alternative to coloured indicators for sighted pupils. A simple phone app has also been developed to detect the endpoint of titrations using a wide range of coloured indicators^13^. The software is freely available, which maximises accessibility, and the endpoint of a given reaction is signalled by sound or vibration. Olfactory changes have also been used to illustrate the concept of adsorption of organic molecules onto activated carbon^14^ and to probe the kinetics of ester formation^15^.

## Challenges and outlook

There have been some exciting advances in making chemistry accessible for pupils with VI and this is by no means a comprehensive review. However, there are numerous experiments and areas of chemistry where accessibility has not been considered. Given the attainment gap in secondary school and the fact that so many pupils with VI feel discouraged when accessing science, it is important that the chemistry community works to make school chemistry accessible. A diverse workforce is one which values different skills, and by opening up the chance to study science to more young people we will be able to solve problems in more inclusive and exciting ways.
